# Is there a role for apolipoprotein E in perinatal brain injury?

**DOI:** 10.1186/s40348-026-00223-6

**Published:** 2026-03-03

**Authors:** Savina Abraham-Pol, Ertan Mayatepek, Mark Dzietko

**Affiliations:** Department of General Pediatrics, Neonatology and Pediatric Cardiology, University Hospital Düsseldorf, Heinrich-Heine-University, Düsseldorf, Germany

**Keywords:** Neonatal, Brain injury, Apolipoprotein, Hypoxia-ischemia, Hemorrhage, Inflammation, Stroke

## Abstract

Perinatal brain injury remains a major cause of neonatal morbidity and lifelong neurological disability. Despite advances in neonatal care, the molecular determinants that modulate vulnerability and recovery in the immature brain remain poorly defined. Genetic variation is increasingly recognized as a contributor to the heterogeneous susceptibility observed among affected infants. Among the genes implicated in neurodevelopmental outcomes, apolipoprotein E (ApoE) has emerged as a key regulator of lipid metabolism, neuroinflammatory signaling, and neuronal repair in the central nervous system.

This systematic review examines clinical studies linking APOE genotype to the occurrence, severity, and outcome of perinatal brain injury, including intraventricular hemorrhage, hypoxic–ischemic encephalopathy, and perinatal stroke, and integrates these findings with a narrative synthesis of experimental and mechanistic literature. Available human data remain limited and heterogeneous. While some studies suggest that the ε2 and ε4 alleles may be associated with increased susceptibility to severe injury and poorer outcomes, findings are inconsistent and require independent replication.

Preclinical studies further demonstrate that ApoE modulates glial activation, lipid and cholesterol transport, blood–brain barrier integrity, and neuronal survival following hypoxic–ischemic and inflammatory insults. Isoform-specific effects, especially associated with ApoE4, appear to exacerbate neuroinflammatory and vascular dysfunction. However, despite converging evidence from animal models and adult neurological disease, ApoE-dependent mechanisms in the developing brain remain insufficiently explored.

Overall, this review highlights APOE genotype as a plausible contributor to vulnerability following perinatal brain injury and underscores the need for large, well-characterized neonatal cohorts and developmentally appropriate mechanistic studies to inform future neuroprotective strategies.

**Supplementary Information:**

The online version contains supplementary material available at 10.1186/s40348-026-00223-6.

## Introduction

### Perinatal Brain Injury – Definition and common types

#### Definition 

Perinatal brain injury (PBI) encompasses a diverse group of brain insults that occur during the fetal and perinatal period, typically defined as extending from approximately 20 weeks of gestation to the first 28 postnatal days. This developmental window is marked by rapid neurodevelopmental processes, making the brain particularly vulnerable to injury [88]. PBI can result from a variety of mechanisms, including hypoxia-ischemia, hemorrhage, infection, inflammation, or disturbances in cerebral blood flow regulation. These injuries are not uniform; they differ significantly in etiology, timing, and affected brain regions, and they contribute substantially to neonatal mortality and long-term neurodevelopmental disability [24, 68].

The global burden of perinatal brain injury is considerable. In 2020, approximately 13.4 million babies were born preterm (gestational age < 37 weeks), accounting for nearly 10% of all live births, and these infants represent the majority of those at highest risk for PBI ([69]; WHO, [90]). Preterm birth, especially before 32 weeks of gestation, remains a leading cause of death in children under five years of age and is associated with a wide range of chronic morbidities, including cerebral palsy, epilepsy, intellectual disabilities, sensory deficits, and behavioral disorders [71]. Even in infants born at term, perinatal hypoxia or stroke can result in profound and lifelong impairments [76].

#### Common types of PBI

**Fig. 1:**
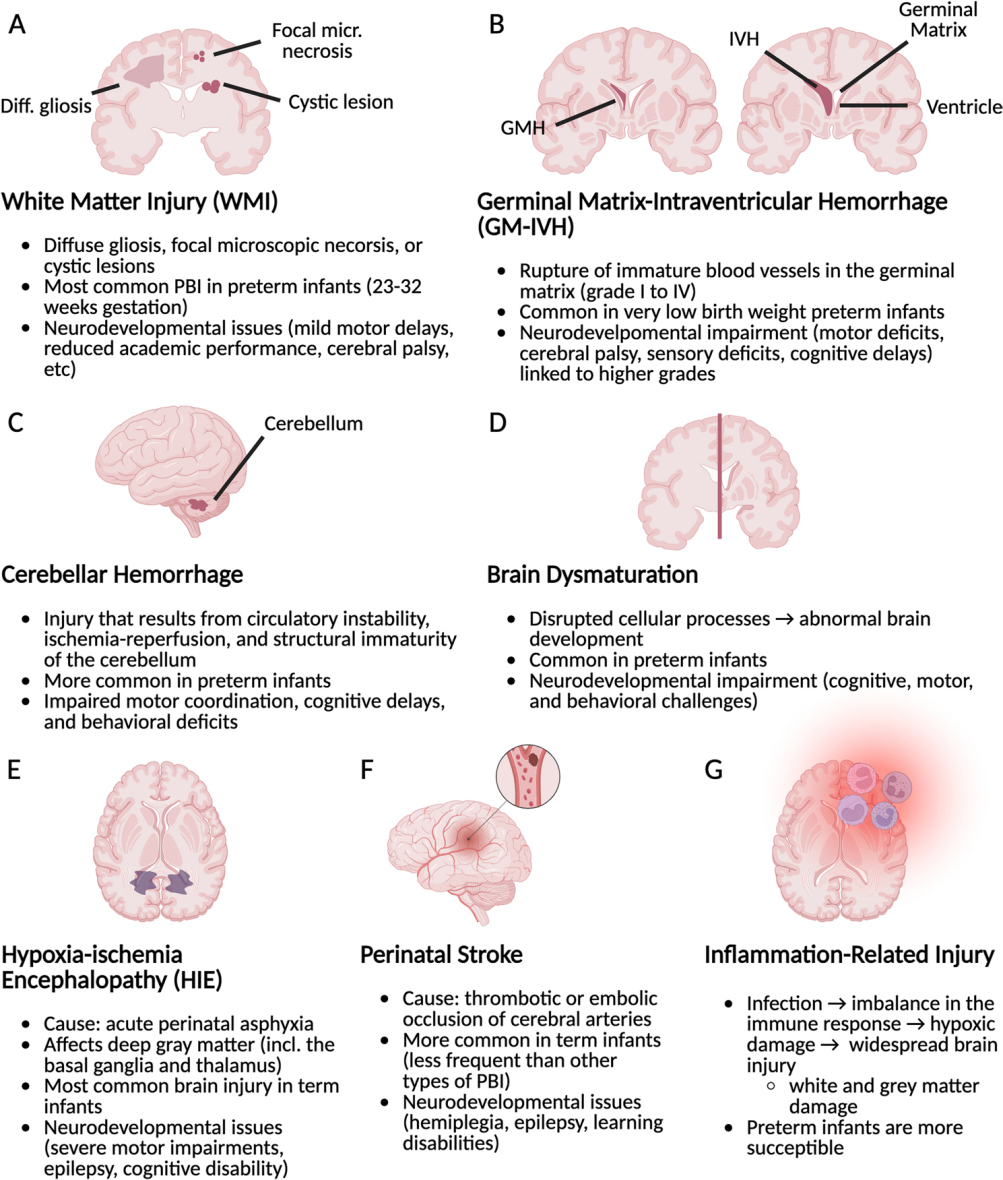
Summary of common types of perinatal brain injury (PBI), their causes, most affected population, and adverse effects. **A** White Matter Injury (WMI). **B** Germinal Matrix-Intraventricular Hemorrhage (GM-IVH). **C** Cerebellar Hemorrhage. **D** Brain Dysmaturation. **E** Hypoxia-Ischemia Encephalopathy (HIE). **F** Perinatal Stroke. **G** Inflammation-Related Injury

PBI encompasses a heterogeneous group of conditions whose presentation depends largely on gestational age, brain maturation, and underlying pathophysiological mechanisms [24, 68, 88]. Preterm and term infants exhibit distinct injury patterns due to differences in vascular architecture and cellular vulnerability (Fig. 1).

In preterm infants, the most frequent forms of injury include white matter injury (WMI), germinal matrix–intraventricular hemorrhage (GM-IVH), cerebellar hemorrhage, and brain dysmaturation. WMI, particularly prevalent between 23 and 32 weeks of gestation, arises from the vulnerability of pre-oligodendrocytes to hypoxic-ischemic and inflammatory insults and is associated with a broad spectrum of long-term neurodevelopmental impairments [24, 47]. GM-IVH results from rupture of fragile germinal matrix vessels, typically within the first days after birth, and higher grades are strongly associated with adverse motor and cognitive outcomes [68]. Cerebellar hemorrhage and brain dysmaturation, increasingly recognized with advanced neuroimaging, further contribute to long-term motor, cognitive, and behavioral deficits in preterm populations [47].

In term and near-term infants, hypoxic-ischemic encephalopathy (HIE) and perinatal arterial ischemic stroke represent the most common forms of PBI. HIE results from acute perinatal asphyxia and primarily affects deep gray matter structures, whereas perinatal stroke is often linked to thromboembolic events and may lead to hemiplegia, epilepsy, and cognitive impairments [24, 68]. More recently, neonatal encephalopathy has been adopted as a broader clinical framework encompassing multiple etiologies, including but not limited to hypoxic-ischemic injury [16, 33, 63].

Across gestational ages, inflammation and infection-related mechanisms play a central role in exacerbating brain injury. Maternal infection, systemic immune activation, and dysregulated myeloid cell responses can sensitize the developing brain to injury and influence both acute damage and long-term repair processes [42, 66, 68, 78].

### ApoE genotype as a genetic risk factor for PBI

Apolipoprotein E (ApoE) is a critical protein involved in lipid metabolism and neuronal health, particularly in the central nervous system (CNS). It plays a pivotal role in the transport of cholesterol, myelination, synaptic plasticity, and amyloid-beta clearance [29].The ApoE gene exists in three isoforms, ε2, ε3, and ε4, that differ in their structural properties and functional implications [59]. The presence of the ε4 allele is associated with an increased risk of neurodegenerative diseases such as Alzheimer’s disease (AD), while ε2 is linked to a lower risk of such conditions [81]. However, much of the research on ApoE has focused on its role in aging and neurodegeneration, particularly in adults, with relatively little attention given to its potential involvement in perinatal cerebrovascular events and PBI.

Despite the importance of ApoE in the adult brain, the study of its relationship with perinatal cerebrovascular events, such as HIE, perinatal stroke, and IVH, remains mainly underexplored. Although ApoE genotype has been shown to influence recovery from cerebrovascular events in adults [83], evidence regarding its impact in the neonatal period is sparse, and existing cohort studies are limited. The limited scope and fragmentation of existing neonatal studies have hindered a comprehensive understanding of how ApoE genotype influences susceptibility to and recovery from PBI.

ApoE has been implicated in neurological outcomes in several cohort studies of perinatal cerebrovascular events. For example, some studies have identified correlations between ApoE genotype and the incidence of severe IVH in preterm infants, suggesting that the ApoE ε2 and ε4 alleles may serve as important risk factors [26]. Similarly, studies have shown that ApoE genotype may influence neurodevelopmental outcomes, including the development of cerebral palsy (CP) in infants with a history of IVH [46]. However, the availability of other similar studies is limited.

A further critical gap lies in the limited understanding of the molecular mechanisms through which ApoE influences perinatal brain injury. While the protein’s role in lipid transport and neuronal repair is well-established, its specific functions in the developing brain and during PBI remain largely unexplored. Differences in brain maturation between neonates and adults, combined with the unique vulnerabilities of the immature brain, may contribute to distinct ApoE-mediated pathways during perinatal brain injury.

In light of these knowledge gaps, the primary objective of this review is to synthesize the available evidence on the relationship between ApoE genotype and PBI. Specifically, this review examines associations between ApoE genotype and the occurrence, severity, and recovery from perinatal cerebrovascular events, with an emphasis on potential underlying molecular mechanisms. By consolidating the existing literature, this review aims to critically evaluate whether ApoE genotype may represent a potential marker of vulnerability, helping to identify at-risk populations that may benefit from early intervention and ultimately improving prevention and treatment strategies for PBI.

Accordingly, this review first summarizes the available clinical evidence linking ApoE genotype and PBI, and subsequently discusses experimental and adult literature to explore potential mechanistic pathways.

## Methods: Search strategy and review methodology

A systematic literature search was conducted to identify studies investigating the association between APOE genotype and PBI, including IVH, HIE, and perinatal stroke. This component of the review was performed in accordance with PRISMA guidelines [70] and is presented in Sect. 4. The PRISMA flow diagram was generated using the PRISMA 2020 Shiny application [37].

PubMed was searched using predefined terms combining “Apolipoprotein E” or “ApoE” with perinatal and neonatal descriptors and cerebrovascular outcomes. Studies were eligible if they were original cohort or case–control studies published in English and examined associations between APOE genotype and PBI-related clinical outcomes. Studies including neonates, infants, or children with perinatal-acquired brain injury were considered eligible, even if outcome assessments extended into later childhood. Studies restricted to adult-onset cerebrovascular disease were excluded. Where applicable, we noted whether cohorts included only survivors or broader birth cohorts.

Title and abstract screening as well as full-text assessment were performed independently by two reviewers, and discrepancies were resolved by discussion.

Given the limited number of eligible clinical studies and their heterogeneity in design, population age at outcome assessment, and outcome definitions, a quantitative meta-analysis was not feasible. Therefore, findings were synthesized narratively, focusing on direction and consistency of reported associations. Due to the small number of studies and variability in reporting, no formal risk-of-bias tool was applied; however, key methodological features (study design, cohort size, age at follow-up, and adjustment for confounders) were critically appraised and discussed in the text.

Due to the limited availability of perinatal-specific mechanistic data, Sects.  5 and 6 comprise a narrative review of experimental and molecular studies selected based on biological relevance to lipid metabolism, neuroinflammation, and cerebrovascular injury in the developing brain. No formal systematic search was conducted for these experimental and adult studies, which were included to provide biological context and hypothesis-generating mechanistic insight. This mixed systematic–narrative approach was chosen to integrate clinical evidence with mechanistic insight in a field where human perinatal data remain sparse.

## Results: Correlation between ApoE genotype and perinatal cerebrovascular events

**Fig. 2:**
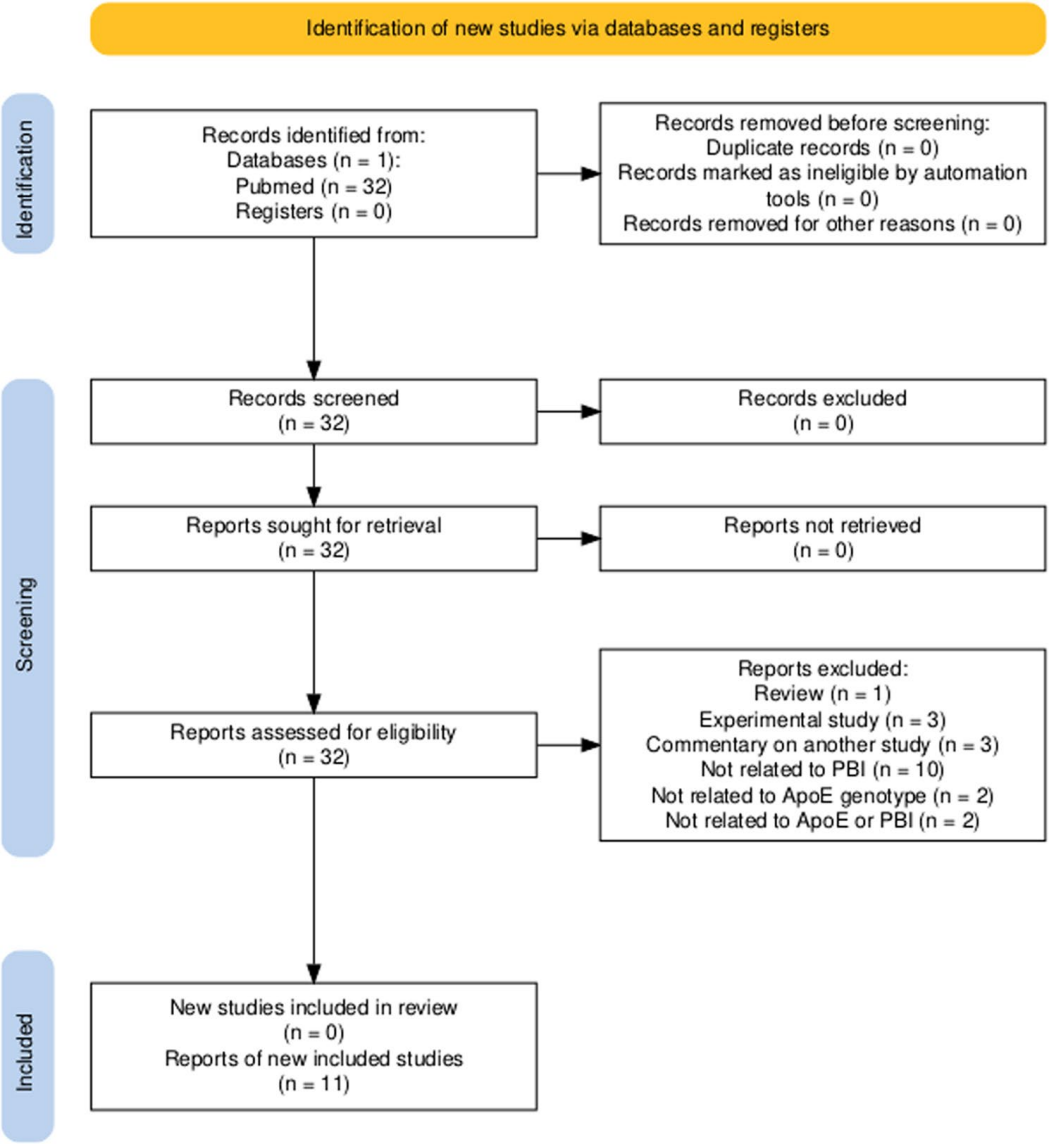
PRISMA flow diagram of study selection. PRISMA 2020 flow diagram illustrating the identification, screening, eligibility assessment, and inclusion of studies evaluating the association between apolipoprotein E (ApoE) genotype and perinatal cerebrovascular events. A total of 32 records were identified through PubMed. After screening and eligibility assessment, 11 studies met the inclusion criteria and were included in the qualitative synthesis. Reasons for exclusion at the eligibility stage are indicated

The systematic search identified 32 records. After screening titles and abstracts, 21 articles were excluded due to lack of relevance to perinatal brain injury or absence of ApoE-related outcomes. Eleven studies met the inclusion criteria and were included in the qualitative synthesis (Fig. 2). These comprised cohort or case–control studies examining associations between ApoE genotype and perinatal or pediatric cerebrovascular outcomes.

Some of the included studies investigated the relationship between ApoE levels or ApoE genotype and perinatal deaths. First, Mc Neil et al., [61] , observed an increase in ApoE levels in neurons during immunohistochemical analysis of brain samples from five Scottish infants who died with perinatal hypoxic-ischemic injury (HII). After that, Becher and colleagues [9] analyzed the distribution of ApoE alleles among a larger sample of Scottish perinatal deaths (186 stillborn infants and 67 liveborn infants with deaths up to 7 days of age). Compared to healthy neonates, an over-representation of the ε2 and ε4 alleles was observed in perinatal deaths. However, the observed tendency was not statistically significant [9].

Other studies focused on the relationship between ApoE genotype and cerebral palsy. However, the studies did not specify the cause of cerebral palsy and none of them showed a clear, statistically significant relationship between ApoE genotype and neurodevelopmental outcomes. Initially, the study of Blackman et al., [12] indicated a possible protective role of the ε4 allele after observing that among a group of 158 children with cerebral palsy (mean age 9.1 ± 9.3 years) the individuals with at least one ε4 allele had lower severity symptoms. However, the results were not statistically significant [12]. Subsequently, Korja et al., [49] and Blackman et al., [11] investigated cohorts of premature infants with very low birth weight. While Blackman et al., observed a relatively high prevalence of the ApoE ε4 allele in their cohort (32% carried at least one ε4 allele), neither study found a statistically significant association between ApoE genotype and short- or mid-term neurodevelopmental outcomes [11, 49].

In total, only six studies have explored the correlation between the ApoE genotype and the frequency, severity, or recovery from cerebrovascular events in children (summarized in Table 1). Two of these studies included children up to 18 years of age [6, 21]. Both studies focused on ischemic stroke, and neither found a significant correlation between the ApoE genotype and the incidence of ischemic stroke. However, in the study by Balcerzyk et al., [6], a higher frequency of the ApoE4/4 genotype was observed in the stroke group, although this trend was not statistically significant, possibly due to the small sample size (*n* = 72). The remaining four studies focused on perinatal cerebrovascular events.

**Table 1:** Summary of cohort and case/control studies exploring the correlation between ApoE genotype and the frequency, severity, or recovery from cerebrovascular events in children

Study	Neurovascular event	Case group	Control group	Key Findings	Conclusion	
Balcerzyk et al., [6]	Ischemic stroke in childhood	Polish children (6 months − 18 years) with ischemic stroke (*n* = 72)	Polish children (age- and sex-matched) without ischemic stroke (*n* = 71)	Higher frequency of ApoE ε4/ε4 genotype in the stroke group, but non-significant	No significant relationship found between ApoE genotype and ischemic stroke incidence in children	
Gelfand et al., [31]	Perinatal Arterial Ischemic Stroke (AIS)	Non-Hispanic white Infants with perinatal (in utero - up to 28 days after birth) AIS (*n* = 13)	Healthy non-Hispanic white neonate (up to 28 days after birth) Infants (*n* = 86)	Higher allelic frequency of ε4 and lower allelic frequency of ε3 in infants with perinatal AIS	ApoE ε4 is associated with increased risk of perinatal AIS	
Cotten et al., [22]	Recovery* after neonatal Hypoxic-Ischemic Encephalopathy (HIE) *Recovery: neurodevelopmental outcome at 18–22 months	Cohort study: Surviving infants with moderate or severe neonatal HIE (with gestational age of at least 36 weeks and birthweight above 1800 g) (*n* = 129)		No evidence for an association of the ApoE ε3/ε3 genotype with improved neurodevelopmental outcome or for an association between presence of either the ApoE ε2 or ε4 allele and increased risk for neurodevelopmental disability (Limitation: only survivors tested)		
Coen Herak et al., [21]	Perinatal and Childhood Arterial Ischemic Stroke (AIS)	Caucasian Children (up to 18 years) with AIS (*n* = 73)	Healthy age-, race-, and sex-matched children (*n* = 100)	No significant effect of ApoE genotype on ischemic stroke risk (Study focused on coagulation factors, not much emphasis on ApoE)		
Dzietko et al., [26]	Perinatal Intraventricular Haemorrhage (IVH)	Cohort study: Risk population for IVH: Infants born preterm (gestational age between 22 and 31 weeks + 6 days) with birthweight below 1500 g (*n* = 7952). All enrolled from German neonatal intensive care units participating in the German Neonatal Network (GNN) between 2009 and 2014.		In a risk population for IVH, ApoE ε2 and ε4 alleles are associated with more severe haemorrhage. Dose-response relationship observed: higher incidence of severe haemorrhage in infants with 2 copies of the ε2 and/or ε4 alleles.		ApoE ε2 and ε4 alleles are relevant IVH risk factors in infants born preterm with very low birth weight
Humbert et al., [46]	Long-term recovery* from perinatal Intracerebral Haemorrhage (ICH) *Recovery: neurodevelopmental outcome at 5–6 years	Cohort study: Part of cohort from Dzietko et al., [26]. Subgroup: Infants born below 30 weeks or with birthweight below 1000 g (*n* = 2215)		ApoE ε4 is associated with higher degree of Cerebral Palsy (CP) compared to ApoE ε3. The association is higher (8-fold) for infants with less severe ICH (grade I).		ApoE genotype influences recovery after intracerebral hemorrhage in preterm infants. ApoE ε4 allele may worsen recovery even after low grade haemorrahge.

One notable cohort study by Cotten et al., in 2014 [22] did not find an association between the ApoE genotype and mid-term recovery from neonatal HIE. This study included 129 infants with a gestational age of at least 36 weeks and a birth weight above 1800 g who survived moderate or severe neonatal HIE, and their neurodevelopmental outcome was assessed at 18–22 months of age [22]. It is important to note that this study only included surviving infants, which could introduce bias, as certain genotypes may be associated with higher mortality rates during the neonatal period.

On the other hand, in 2013, Gelfand et al., [31] found an increased risk of perinatal arterial ischemic stroke associated with the ApoE ε4 allele. However, the sample size was extremely small (*n* = 13), which raises concerns about the reliability and generalizability of these findings.

In general, all four studies presented above focused on small cohorts or case groups, which may have contributed to the often non-significant or contradictory results. As a result, it is difficult to draw any definitive conclusions about the impact of different ApoE alleles on the occurrence, severity, or mid- to long-term outcomes of perinatal cerebrovascular events. Additionally, these studies vary significantly in terms of factors such as the age of onset of the event, or gestational age or birth weight of the infants, further complicating the interpretation of the results.

In contrast to early studies, which were predominantly small and underpowered, Dzietko et al., [26] conducted a much larger multicenter cohort study that provided valuable insights into the role of the ApoE genotype in perinatal cerebrovascular events. Their study involved a high-risk population for IVH: extremely and very preterm infants (22 < 32 weeks gestational age) with a birth weight below 1500 g. The cohort comprised 7952 infants from 53 German neonatal intensive care units (NICUs) born between 2009 and 2013. The clinical data was collected through the German Neonatal Network (GNN), a nationwide registry that compiles comprehensive neonatal outcome data from multiple NICUs across Germany. This registry plays a pivotal role in facilitating large-scale, multi-center studies on neonatal health outcomes.

Among the infants in this cohort, 1456 suffered from neonatal IVH, defined as any bleeding into the cerebral germinal matrix or the ventricles, and classified from grade I to IV. In the total cohort of 7952 infants, 5075 (63.8%) carried the ApoE3 genotype, 965 (12.1%) ApoE2, and 1912 (24.0%) ApoE4. The study found a significant correlation between the ApoE alleles ε2 and ε4 and the occurrence of severe IVH (grades III and IV). Severe IVH occurred in 281/5075 (5.5%) of ApoE3 carriers, compared to 75/965 (7.8%) of ApoE2 carriers and 146/1912 (7.6%) of ApoE4 carriers. Furthermore, a dose-response relationship was observed, with the incidence of severe hemorrhage being higher in infants who had more than one copy of the ε2 and/or ε4 alleles (7.6% in infants with one polymorphism and 8.7% in those with two polymorphisms, compared to 5.5% in ApoE3/E3 infants). On top of that, the incidence of more severe IVH (grades III to IV) was significantly higher in infants with the ApoE4 genotype compared to ApoE3 (38.6% vs. 31.8% among infants with IVH). These associations remained even after adjusting for other known risk factors for IVH, such as gestational age and antenatal steroid treatment. Based on these findings, the study concluded that the ApoE ε2 and ε4 alleles are relevant risk factors for IVH in preterm infants [26].

Additionally, the long-term neurological outcomes of a subgroup of this cohort were assessed in a follow-up study by Humberg et al., [46]. The follow-up included 2215 infants born with a gestational age below 30 weeks or a birth weight below 1000 g, and their neurodevelopmental outcomes (defined in terms of the level of cerebral palsy; CP) were assessed at 5–6 years of age. In this follow-up cohort, 1416 (63.9%) carried ApoE3, 272 (12.3%) ApoE2, and 527 (23.8%) ApoE4. Of the 2215 infants, 363 (16.4%) had suffered from neonatal IVH. When examining the group of infants with a history of neonatal IVH, the ApoE alleles ε2 and ε4 were associated with an increased prevalence of CP compared to the ApoE3 allele, with the ε4 allele being associated with the highest prevalence (29% [31/107], compared to 23.8% [15/63] in ε2 carriers and 13.2% [33/250] in ε3 carriers). However, after adjusting for known predisposing factors, only the ApoE ε4 allele had a significant effect on the prevalence of CP (OR 2.83, 95% CI 1.46–5.49). Interestingly, the negative effect of the ApoE4 genotype was most pronounced in infants with grade I IVH, where the prevalence of CP was 15.4% (6/39) compared to 1.9% (2/105) in ApoE3 carriers [46].

## Discussion

The present systematic review identified a small number of heterogeneous studies examining associations between APOE genotype and PBI, with predominantly inconsistent or non-significant findings. Although neonatal data remain sparse and largely inconsistent, one large multicenter cohort study reported isoform-specific associations, particularly involving the ε4 allele, which require independent validation. Importantly, the majority of studies reporting statistically significant associations between ApoE genotype and PBI outcomes have originated from a single research group, underscoring the need for independent replication in large, well-characterized cohorts.

To appropriately interpret these findings and avoid underestimating their potential relevance, it is essential to consider the extensive body of preclinical and adult literature demonstrating robust ApoE isoform–dependent effects on cerebrovascular integrity, lipid metabolism, inflammation, and neurovascular resilience. The following sections therefore integrate mechanistic and adult clinical evidence to provide biological context for the neonatal findings.

### Apolipoprotein E: the main apolipoprotein of the central nervous system

**Fig. 3:**
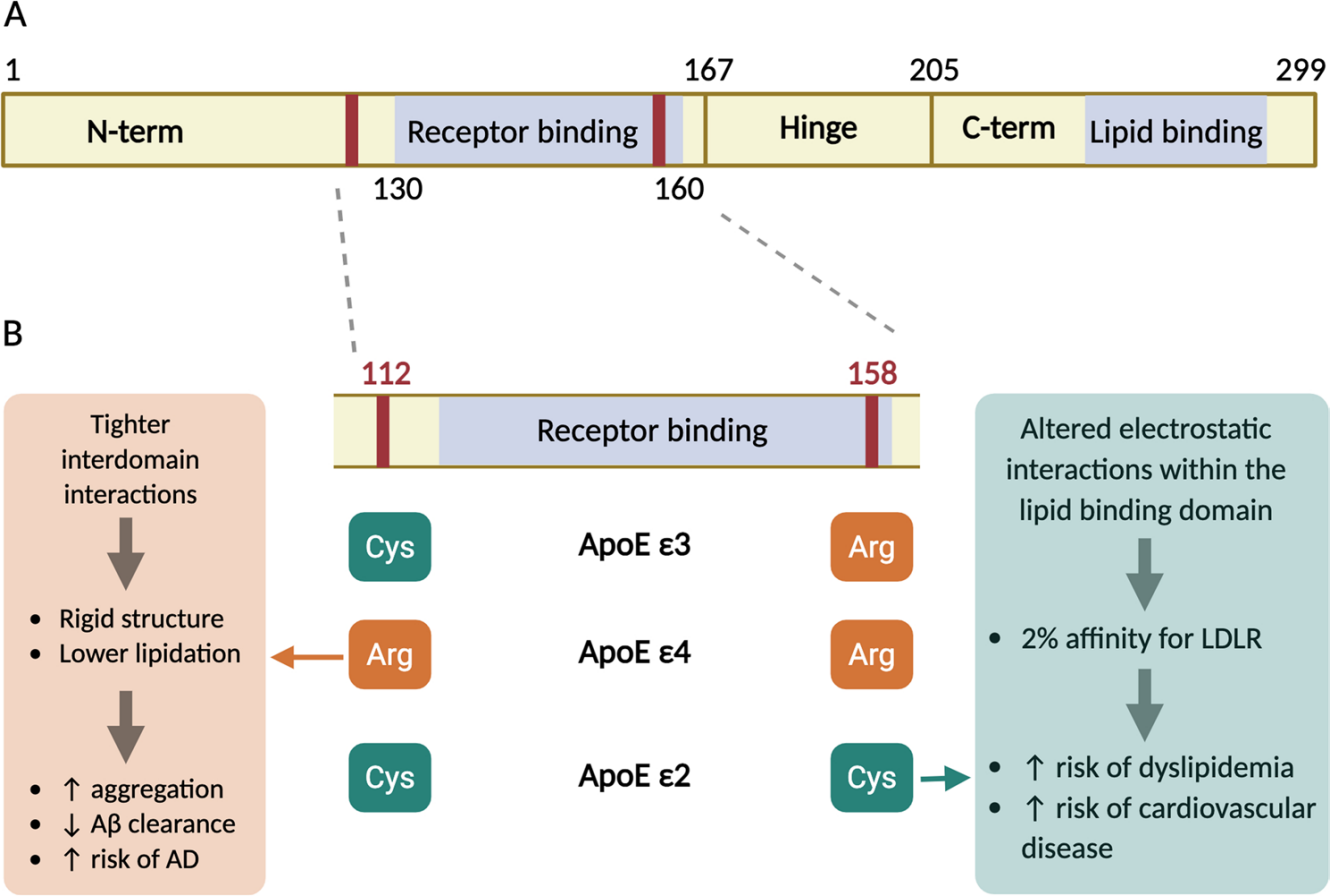
Human Apolipoprotein E sequence and common isoforms. **A** Schematic domain structure of ApoE. **B** Amino acid differences among the major human ApoE isoforms (ε2, ε3, ε4) and consequences on the structure and function of the protein

ApoE is a 34 kDa and 299 amino acid apolipoprotein (Fig. 3A) that was first isolated from human plasma-derived very-low-density lipoproteins (VLDL) in 1975 [87]. It is a component of multiple lipoproteins and has high affinity for diverse cell surface receptors and extracellular matrix components, therefore acting as a key factor in lipid transport and metabolism [52, 58, 65]. ApoE is synthetized by multiple cell types and is found in both the CNS and the periphery. However, the ApoE in the brain and in the periphery comprise two different pools, since peripheral ApoE is not able to cross the blood-brain barrier (BBB) into the CNS [29, 52, 81]. Even though most of the total ApoE in the body (75%) is synthetized by the hepatocytes and corresponds to the peripheral pool, ApoE is the most abundant apolipoprotein in the brain, where it is mostly synthetized by astrocytes and also oligodendrocytes and microglia to a lesser extent. Furthermore, it is also upregulated in neurons after injury or stress [29, 52, 58].

In the CNS, astrocytes represent the main source of ApoE under physiological conditions and act as a central hub for ApoE lipidation and secretion. Astrocyte-derived ApoE is assembled into high-density lipoprotein (HDL)-like lipoprotein particles in an ATP-binding cassette transporter A1 (ABCA1)-dependent manner, which is essential for ApoE stability and lipid loading [44, 89]. These ApoE-containing particles are released into the interstitial fluid and cerebrospinal fluid and taken up by neighboring cells through members of the low-density lipoprotein (LDL) receptor family, including low-density lipoprotein receptor (LDLR) and LDL-Receptor Related Protein 1 (LRP1), supporting lipid and cholesterol redistribution within the brain and establishing ApoE as a largely paracrine regulator of CNS lipid homeostasis [53, 73].

In the CNS, ApoE supports neuronal health and cognitive function through different mechanisms. For example, it participates in the transport of cholesterol, which is highly required for neuronal health as component of the cell membrane, and also needed for myelination, synapses and neuronal plasticity. Furthermore, it can interact with amyloid-beta (Aß), contributing to the clearance and degradation of Aß plaques in an isoform dependent manner [52].

Beyond neurons, ApoE exerts lineage-specific functions in other glial cells. In microglia, ApoE expression is low under homeostatic conditions but is strongly upregulated following injury and neurodegeneration, where it acts as a key component of the TREM2–APOE pathway that drives the transition to disease-associated microglial states and regulates immune–metabolic responses (Keren-Shaul et al., 2017; Krasemann et al., 2017). In the oligodendrocyte lineage, ApoE-dependent lipid availability is critical for differentiation and myelin maintenance, and impaired ApoE-mediated lipid transport, particularly associated with the APOE4 isoform, has been linked to defective oligodendrogenesis and increased myelin vulnerability [7, 13].

In the periphery, ApoE assists the transport of triglycerides and cholesterol to peripheral tissues, functioning primarily as a ligand mediating lipoprotein uptake through interactions with cell surface receptors, LDLR and LRP1, as well as extracellular matrix components such as heparan sulfate proteoglycans (HSPGs) [32, 57, 65]. These receptor-mediated mechanisms parallel ApoE’s role in the central nervous system, where similar interactions regulate lipid transport and cellular uptake.

While some of these functions are conserved in the CNS, important differences exist. Due to the presence of the BBB, brain cholesterol metabolism is largely autonomous and relies on locally produced ApoE-containing HDL-like particles. In this context, ApoE-mediated lipid transport primarily supports cellular maintenance, membrane remodeling, synapse formation, and myelin synthesis rather than systemic lipid distribution [53, 73].

Although much of the mechanistic insight into ApoE biology derives from studies in AD, the cellular processes regulated by ApoE (including glial lipid metabolism, myelination, and injury-induced glial activation) are fundamental to CNS injury responses across the lifespan. The developing brain is characterized by high lipid demand and dynamic astrocyte–microglia reactivity during hypoxic–ischemic and inflammatory insults, processes in which ApoE expression and function are strongly modulated following injury [29, 73]. In this context, ApoE genotype may influence susceptibility to and recovery from PBI by modulating conserved pathways of lipid redistribution and glial injury responses.

#### Apolipoprotein E – structure

ApoE is synthetized at the endoplasmic reticulum and secreted through the Golgi apparatus. Its immature form has 317 amino acids and contains an 18 amino acid signal peptide that is cleaved during maturation, resulting in a mature glycosylated 299 amino acid protein with a molecular weight of 34 kDa [59].

ApoE is divided into three structural domains: an N-terminal domain (residues 1-167), a hinge region (168–205), and a C-terminal domain (206–299) (Fig. 3A). The N-terminal domain is composed by four antiparallel helices, while the hinge region and C-terminal domain contain two and three helices respectively. Overall, protein displays and helix-bundle structure in which the N-terminal domain is localized between the hinge region and the C-terminal domain [20, 29, 59].

The receptor-binding region, which preferentially binds LDLR and LRP1, is localized within the N-terminal domain, spanning the residues 130–160. On the other hand, the C-terminal domain is responsible for the lipid-binding activity of ApoE. In the tertiary structure (Fig. 3A), the N- and C-terminal domains tightly interact with each other through H-bonds and salt-bridges between their polar residues. The C-terminal domain and hinge region are not able to fold on their own, therefore the interdomain interactions are essential in a two-step folding process, in which the N-terminal domain is folded first and acts as a template to assist the folding of the hinge region and C-terminal domain. Besides assisting the protein folding process, the interactions between domains help stabilize the C-terminal domain, which has multiple exposed hydrophobic residues responsible for binding lipids, HSPG, and Aß peptides [20].

The structure of ApoE, as well as the conformational changes associated to lipidation, ensure that only fully lipidated ApoE is able to bind to LDLR. When the protein is not lipidated, it displays an inactive helix-bundle conformation in which the interactions between the N- and C-terminal domains shield the receptor-binding region, hindering its interaction with lipoprotein receptors. As soon as the C-terminal domain interacts with lipids, it opens up leading to an intermediate conformation in which the C-terminal domain is partially lipidated and the receptor-binding region is only partially exposed (but still not able to bind to receptors). Only when the C-terminal domain is fully lipidated, the area between the C- and N-terminal domains fully opens, leading to an active conformation in which the C- and N-terminal domains form two lobes linked by the hinge region. In this conformation both lobes interact with the surface of lipoproteins, and the receptor-binding region is fully exposed and available for interaction with receptors [20].

Importantly, subtle sequence differences among ApoE isoforms influence these interdomain interactions and folding dynamics, with downstream consequences for lipidation efficiency and cholesterol metabolism, as discussed below.

#### Apolipoprotein E – isoforms and specific effects on structure and function

In humans, there are three major ApoE isoforms (ApoE2, ApoE3 and ApoE4) that are encoded by three different alleles. Those isoforms are different from each other only by an amino acid exchange at the positions 112 and 158 [59]. The ApoE3 allele, which is the most common allele (present in 65–70% population) and considered wild type, has a cysteine residue at the position 112 and an arginine at the position 158. On the other hand, the ApoE4 (found in 15–20% of population) and the ApoE2 (frequency of 8–10%) alleles, have either arginine or cysteine in both residues respectively (Fig. 3B) [59].

Even though the difference in sequence among common ApoE isoforms is small, it can have an impact on the structure and function of the protein. For example, in ApoE2, the presence of a cysteine instead of an arginine at position 158 alters the electrostatic interactions between the sidechains of the residues at the receptor-binding region, disrupting the normal salt bridge formed between the residue 150 and the LDLR. As a result, ApoE2 has just around 2% affinity for LDLR compared to ApoE3 or 4. However, it can still bind HSPG or other receptors ([29]; Marais, 2021; [59]). As a result, the ApoE2 genotype is associated with higher risk of dyslipidaemia and cardiovascular disease (risk that is further increased by homozygotes) (Fig. 3B) [56, 86].

On the other hand, in ApoE4, the presence of arginine instead of cysteine at position 112 favors ionic interactions between the N-terminal and C-terminal domains, leading to tighter interdomain interactions and a more rigid overall structure (Fig. 3B). As a consequence, ApoE4 has been observed to preferentially bind to VLDL over HDL in the periphery. However, in the CNS, ApoE4 displays lower lipidation compared to ApoE2 and ApoE3, which can result in a higher tendency to form aggregates and a lower capacity for ß amyloid clearance [29]. Therefore, ApoE4 is the highest genetic risk factor for sporadic AD [81]. Some of the proposed mechanisms linking ApoE4 to AD are the reduced clearance of amyloid ß plaques, increased proteolysis of ApoE4 (which leads to proteotoxic stress), or modulation of inflammatory molecules like some interleukins [29].

Thus, although ApoE isoforms differ by only two amino acids, these substitutions have distinct effects on interdomain interactions and folding dynamics. ApoE2 primarily affects receptor-binding efficiency without major changes in folding stability, whereas ApoE4 promotes tighter N–C terminal domain interactions, leading to altered lipidation efficiency. These isoform-specific structural properties directly influence cholesterol transport by modulating lipoprotein preference, receptor engagement, and lipid redistribution in both the periphery and the CNS.

Having outlined the structural and functional characteristics of ApoE, the following section examines experimental studies that directly investigate its role in hypoxic–ischemic and related injury models.

### Mechanistic insight from preclinical studies

#### Animal studies

Few studies have explored the molecular mechanisms linking ApoE to cerebrovascular events such as hemorrhage or hypoxia-ischemia in the neonatal brain. One of the first studies to investigate this relationship was conducted by Lendon et al., [51], who used an animal model of neonatal hypoxia-ischemia (HI) involving ApoE knock-out mice (ApoE -/-), which were genetically engineered to express either human ApoE ε3 or ε4 isoforms (ApoE ε3+/-, ApoE ε4 +/-). These animals were subjected to neonatal HI on postnatal day 6 through ligation of the left common carotid artery, followed by a 2-hour recovery period and 45 min of hypoxia in a chamber with 8% oxygen at 37 °C. Afterward, the animals were returned to their dam for up to 14 days before their brains were histologically processed. The results revealed a three-fold increase in ApoE staining in astrocytes, neutrophils, and, occasionally, hippocampal neurons. ApoE levels peaked around 72 h post-injury and returned to baseline within 2 weeks. Importantly, no significant differences in expression levels were observed between the ApoE ε3 and ε4 isoforms, suggesting that ApoE expression is transiently elevated in response to HI injury in a general manner, rather than being isoform specific [51].

In a subsequent study, McAdoo et al., [60] investigated whether the administration of a 17-residue ApoE-mimetic peptide could mitigate CNS injury in a perinatal hypoxia-ischemia rodent model. This peptide, derived from the receptor-binding region (133–149) of ApoE, had previously demonstrated a high affinity for ApoE receptors, neuroprotective effects against excitotoxic injury, and the ability to suppress glial activation. The researchers hypothesized that intrathecal administration of the ApoE-mimetic peptide would provide neuroprotection in the rodent HI model. Postnatal day 7 rat pups underwent carotid artery cauterization and transection, followed by a 90-minute recovery period. The animals then received either ApoE-mimetic peptide or PBS (control) via intracerebroventricular (ICV) injection, and were exposed to a hypoxic chamber (8% oxygen, 37 °C) for 150 min. Seven days after hypoxia (postnatal day 14), the rats were decapitated, and their brains were analyzed. The results showed that ICV administration of the ApoE-mimetic peptide reduced hypoxia-induced hemispheric weight loss in a dose-dependent manner, with the most effective dose (5 mg/kg) resulting in a 49% reduction in hemispheric brain weight loss compared to the PBS control. Additionally, the peptide treatment improved survival, reducing mortality by the study endpoint (postnatal day 14). These findings suggest that ApoE-mimetic peptides, which can interact with ApoE receptors and cross the BBB, may offer neuroprotective and anti-inflammatory effects that help reduce brain injury following perinatal HI [60].

Building on this, a more recent study by Chen et al., [19] explored the role of ApoE in the transport of peroxidized fatty acids (FAs) from neurons to astrocytes for degradation after hypoxia-ischemia. This process, known as neuron-astrocyte coupling of FA metabolism, helps protect neurons from the accumulation of peroxidized FAs, which result from hypoxia-induced oxidative stress. The study found that excessive saturated and peroxidized FAs accumulate in lipid droplets (LDs), which are abnormally present at brain lesion sites. In this study, a perinatal hypoxia-ischemia rodent model was used, in which rats underwent permanent ligation of the right common carotid artery on postnatal day 7, followed by a 1-hour recovery period. The animals were then exposed to hypoxia (8% oxygen) for 30 min and returned to a normal environment. Brain samples analyzed 48 h post-insult showed an increase in ApoE expression, predominantly in astrocytes, as well as an increase in the number and size of LDs, particularly in the hippocampus and cerebral cortex. These results indicate abnormal FA metabolism following hypoxia-ischemia. After a 7-day recovery period, changes in neuronal morphology and an increase in inflammatory markers were also observed, further supporting the hypothesis of ApoE’s involvement in lipid metabolism under stress conditions [19].

In summary, these studies highlight the growing but still incomplete understanding of ApoE’s role in neonatal HI and related cerebrovascular events. Lendon et al., [51] were among the first to show that ApoE expression increases in response to HI, particularly in astrocytes and immune cells, but without differences between the ApoE isoforms, suggesting a general injury response rather than an isoform-specific effect. Building on this, McAdoo et al., [60] found that administering an ApoE-mimetic peptide reduced brain damage and improved survival in a rodent HI model, supporting the potential of ApoE-targeted therapies. More recently, Chen et al., [19] shed light on ApoE’s role in lipid metabolism, showing its involvement in transporting toxic fatty acid byproducts from neurons to astrocytes after HI. Overall, these studies highlight ApoE’s diverse functions in response to hypoxic-ischemic injury, from modulating inflammation and promoting neuronal survival to regulating lipid homeostasis, suggesting it could be a valuable therapeutic target.

#### In vitro cell-based studies

Several cell-based studies have explored the role of ApoE and related mechanisms in neuroprotection, particularly in the context of ischemic injury, neuronal dysfunction, and cellular responses to hypoxia. One of the early investigations into this topic was conducted by Lendon et al., [51], who aimed to determine whether ApoE influences glutamate-mediated excitotoxicity and apoptosis, two key molecular processes that are commonly observed following ischemic insults. In their study, primary neurons were cultured from embryonic day 14 ApoE wild-type (ApoE+/+) and knock-out (ApoE-/-) mice. These neurons were incubated with ApoE for 12 h before being exposed to the glutamate receptor agonist N-methyl-D-aspartic acid (NMDA) or the apoptotic drug staurosporine. The experiment utilized both endogenous ApoE, derived from primary astrocytes, and human ApoE isoforms (ε2, ε3, and ε4) isolated from human plasma HDL particles. While endogenous astrocyte-derived ApoE did not influence the survival of neurons after these treatments, human HDL-derived ApoE significantly reduced drug-induced cell death in ApoE-/- neurons. This finding suggests that ApoE derived from human HDL has neuroprotective effects in neurons lacking ApoE, although interestingly, no significant differences were observed between the ApoE isoforms (ε2, ε3, and ε4). This indicates that the protective effects of ApoE in these experiments were not isoform-specific, highlighting a general neuroprotective role for ApoE that may extend beyond its isoform differences in some contexts [51].

In 2012, Kysenius et al., [50] investigated the role of PCSK9, a secreted protease that regulates cholesterol metabolism through its interaction with the LDL receptor family members, including VLDLR and ApoER2. PCSK9 has been implicated in various neuronal functions, including its role in neuronal apoptosis, particularly during ischemic events. In their study, cerebellar granule neurons (CGNs) isolated from postnatal mice were used to model neuronal apoptosis induced by potassium deprivation. The authors found that knockdown of PCSK9 using RNA interference (RNAi) inhibited the activation of caspase-3 and c-Jun, two key mediators of JNK-dependent apoptosis, thereby reducing neuronal cell death and promoting cell survival. This was associated with increased levels of VLDLR and ApoER2, suggesting that the protective effects of PCSK9 knockdown were mediated by the upregulation of these receptors. Further experiments revealed that inhibition of the JNK and ERK pathways attenuated apoptosis, and combining these inhibitors with PCSK9 RNAi further reduced cell death. These findings indicate that PCSK9 cooperates with the JNK and ERK signaling pathways to regulate neuronal apoptosis and that modulating these pathways could provide potential therapeutic targets for ischemic brain injury [50].

In 2024, Chen et al., [19] extended this line of investigation by studying how ApoE contributes to the transport of FAs from neurons to astrocytes, particularly under ischemic conditions. In their in vitro study, primary neurons from ApoE wild-type and ApoE knock-out rat pups were cultured and subjected to oxygen-glucose deprivation and reperfusion (OGDR), which serves as a model for ischemia. The results revealed a significant increase in lipid peroxidation in neurons exposed to OGDR. Subsequently, recombinant human ApoE isoforms (ε2, ε3, and ε4) were administered to ApoE knock-out neurons. The study found that ApoE isoforms potentiated the transport of peroxidized FAs from neurons to astrocytes and promoted cell survival. Notably, ApoE ε4 was less efficient than ApoE ε2 and ε3 in facilitating FA transport and improving cell survival, suggesting that ApoE isoforms have differential effects in this context. These findings provide important insight into the role of ApoE in fatty acid metabolism and highlight the potential for ApoE isoforms to influence neuronal recovery after ischemic events. Furthermore, this study emphasizes the importance of ApoE’s interaction with astrocytes in maintaining cellular homeostasis and preventing lipid toxicity [19].

In a more recent study, Shrivastava et al., [82] focused on the role of astrocytes and ApoE in the maturation of oligodendrocytes under hypoxic conditions, which are commonly encountered during developmental brain injury. Oligodendrocytes, the glial cells responsible for myelinating neuronal axons, are highly vulnerable to hypoxic stress, particularly during fetal brain development. In this study, human fetal neural stem cells (hFNSCs) were differentiated into astrocytes, which were then co-cultured with premyelinating oligodendrocytes (Mo3.13 cells), a widely used model for studying oligodendrocyte differentiation. The cells were exposed to a controlled hypoxic environment (0.2% oxygen for 48 h), and the results demonstrated that astrocytes under hypoxic stress upregulated genes involved in cholesterol biosynthesis and efflux, including hydroxy-methyl-glutaryl CoA reductase, squalene epoxidase, and ApoE. These upregulated genes facilitated cholesterol transfer from astrocytes to premyelinating oligodendrocytes, which lack the capacity to produce sufficient cholesterol under hypoxia. This cholesterol transfer was found to support the differentiation of premyelinating oligodendrocytes into mature oligodendrocytes, which are essential for myelination. The study highlights the protective role of astrocytes in maintaining the maturation and function of oligodendrocytes under hypoxic stress, suggesting that ApoE-mediated cholesterol transport from astrocytes to oligodendrocytes is critical for supporting myelination in the developing brain [82].

Taken together, these studies highlight the multifaceted role of ApoE in the context of ischemic and hypoxic stress, particularly in the developing brain. ApoE appears to contribute to neuroprotection through several mechanisms, including modulation of lipid metabolism, facilitation of fatty acid and cholesterol transport, and regulation of receptor-mediated signaling pathways. Differences among ApoE isoforms, as well as interactions with other molecules such as PCSK9, may further influence these processes. Overall, these findings support the idea that ApoE plays a central role in maintaining neuronal and glial function after injury, and suggest that targeting ApoE-related pathways could hold therapeutic potential for neonatal brain injury.

While neonatal-specific mechanistic studies remain limited, a substantial body of evidence from adult cerebrovascular and neurodegenerative research provides important insight into isoform-specific vascular and inflammatory mechanisms.

### Evidence from adult cerebrovascular and neurodegenerative disease

Due to the lack of research into the molecular mechanisms linking ApoE genotype and cerebrovascular events in the perinatal context, relevant studies in other contexts are included in this section. While most of this mechanistic evidence comes from aging or AD models, the implicated molecular pathways - such as disruption of BBB integrity, pericyte dysfunction, altered inflammatory responses, Aß accumulation, and dyslipidemia-driven vascular disease - are critically relevant to cerebrovascular pathophysiology and may also play a role in the immature brain. Therefore, this section will examine how ApoE4 contributes to cerebrovascular vulnerability by focusing on studies conducted outside the perinatal context.

#### ApoE4-Induced BBB dysfunction

The BBB plays a central role in maintaining cerebral homeostasis and regulating immune cell trafficking into the brain. Dysfunction of this barrier is an early key event in multiple neurological conditions including AD, vascular cognitive impairment, and ischemic stroke [3, 18]. A body of research has shown that ApoE4 promotes BBB breakdown via both structural and signaling-dependent mechanisms, many of which are likely to contribute to increased susceptibility to cerebrovascular injury.

In an early foundational study, Methia et al., [62] demonstrated that ApoE is required for efficient BBB repair following injury. Using a dye-extravasation approach in ApoE knockout (KO) and wild-type mice, they found a substantial (~ 70%) increase in vascular permeability in ApoE-deficient animals following photochemical injury. Importantly, this effect was not reproduced in LDL receptor KO mice, suggesting a specific, non-lipid-mediated role of ApoE in BBB integrity [62]. These findings are relevant to stroke, where the ability to repair BBB damage after ischemic or haemorrhagic insult is a key determinant of neurological outcome [35].

Hafezi-Moghadam et al., [38] extended these findings by showing that ApoE-KO mice develop progressively increased BBB permeability with age, particularly in postcapillary venules. To dissect the contribution of ApoE from different cellular sources, the authors generated bone marrow chimeric mice by transplanting WT blood cells into ApoE-KO recipients and vice versa. These chimeric animals displayed intermediate levels of BBB permeability, greater than WT but less than ApoE-KO, indicating that ApoE produced by both tissue-resident cells and circulating leukocytes plays a crucial role in maintaining BBB integrity, implicating immune-vascular crosstalk [38]. Since leukocyte infiltration is a critical mediator of post-stroke inflammation and secondary injury [48], ApoE4-induced permeability may prime the cerebrovascular system for a maladaptive immune response during ischemic events.

More direct evidence comes from the study by Teng et al., [85], which examined the effects of ApoE genotype following traumatic brain injury, a model that shares many pathological features with stroke, such as edema, BBB disruption, and neuroinflammation. They found that ApoE4-expressing mice showed significantly increased BBB leakage and brain water content compared to ApoE3 or wild-type mice. This was associated with downregulation of tight junction proteins (occludin, ZO-1) and activation of the NF-κB/MMP-9 pathway, highlighting a direct molecular mechanism by which ApoE4 impairs vascular barrier function [85].

**Fig. 4:**
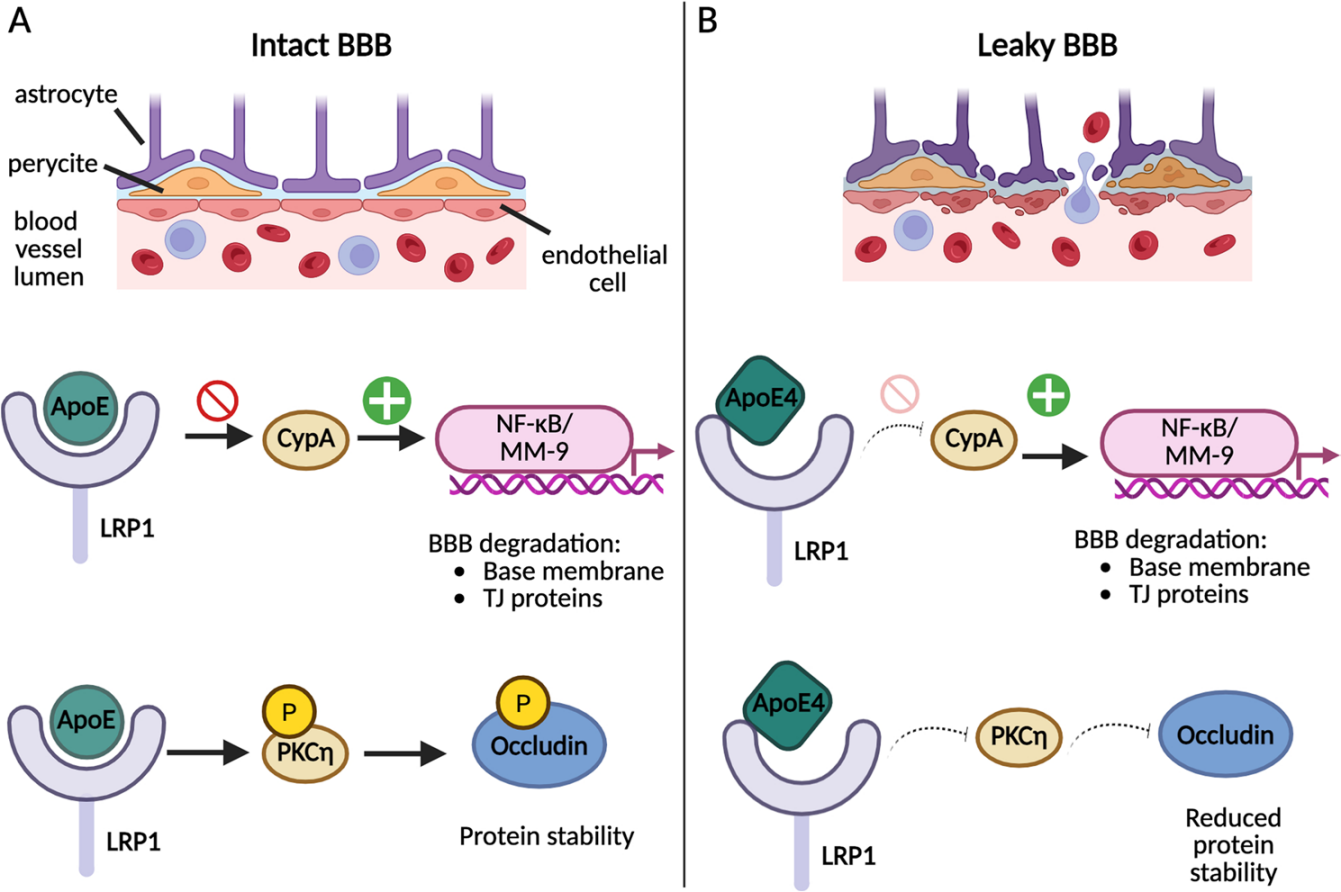
Molecular mechanisms linking ApoE ε4 and blood-brain barrier and pericyte dysfunction. **A** In perycites, by binding LRP1 receptor, ApoE inhibits CypA, which would otherwise activate the NF-kB/MM-9 pathway, leading to degradation of the base membrane and tight junction (TJ) proteins of the blood-brain barrier (BBB). Furthermore, the interaction between ApoE and LRP1 also favors the phosphorylation of the kinase PKCη and the downstream phosphorylation of the TJ protein occludin, contributing to its stability. These mechanisms contribute to a healthy and functional BBB. **B** On the other hand, the human isoform ApoE ε4 interacts weaklier with the receptor LRP1, leading to a decreased inhibition of CypA, which results on more activation of the NF-kB/MM-9 pathway and therefore degradation of the base membrane and TJ proteins of the BBB. In addition, the phosphorylation of PKCη and therefore occludin is also diminished, resulting in its reduced stability. These molecular pathways contribute to the detrimental effect of ApoE ε4 on the stability of the BBB

Perhaps the most detailed molecular analysis of this pathway comes from Bell et al., [10], who demonstrated that ApoE4 fails to suppress cyclophilin A (CypA) expression in brain pericytes due to ineffective binding to LRP1, a key receptor at the neurovascular interface. As a result, CypA activates NF-κB and induces MMP-9, which degrades basement membrane and tight junction proteins, leading to BBB breakdown (Fig. 4A-B). Genetic or pharmacological inhibition of CypA restored BBB integrity in ApoE4-expressing mice [10]. Given that MMP-9 is a known contributor to hemorrhagic transformation after ischemic stroke [36], this mechanism may underlie the higher rates of complications and worse recovery observed in ApoE4 carriers after cerebrovascular events.

While many studies have focused on transcriptional effects, ApoE4 also impairs BBB function through isoform-specific, post-translational mechanisms. Nishitsuji et al., [67] used a co-culture model of endothelial cells and astrocytes to show that ApoE4 astrocytes fail to maintain transendothelial electrical resistance (TEER), a measure for barrier tightness. This was not due to changes in gene expression, but rather to impaired phosphorylation of occludin at threonine residues, a modification necessary for tight junction assembly. They identified a pathway involving LRP1 and protein kinase C eta (PKCη), which was disrupted in ApoE4 astrocytes (Fig. 4A-B). Although this work was performed in vitro, it underscores how ApoE4 can intrinsically weaken the BBB, rendering it more vulnerable to damage during stroke or HI [67].

Further supporting a cerebrovascular role, Alata et al., [1] used brain perfusion assays to show that aged ApoE4 mice exhibit impaired glucose and drug transport across the BBB, despite normal expression of the GLUT1 transporter. These animals also showed shorter, thinner microvessels and basement membrane irregularities, all structural abnormalities that mirror those seen in small vessel disease and chronic ischemic damage [1]. Such vascular remodeling likely exacerbates hypoperfusion and limits the brain’s ability to recover from stroke.

Recent human studies have added critical clinical context to these mechanistic findings. Montagne et al., [64] used dynamic contrast-enhanced MRI to reveal elevated BBB leakage in the hippocampus and medial temporal lobe of cognitively normal APOE4 carriers, independent of amyloid or tau pathology. Similarly, Reas et al., [75] found that increased BBB permeability in the entorhinal cortex was associated with microstructural abnormalities like reduced neurite density and increased free water, even in amyloid-negative individuals. These findings suggest that BBB dysfunction in APOE4 carriers is a primary phenotype rather than a secondary consequence of neurodegeneration, and may predispose the brain to worse outcomes after hypoxic or ischemic events.

Together, these findings converge on the conclusion that ApoE4 significantly compromises BBB integrity through both structural and molecular mechanisms, heightening cerebrovascular vulnerability.

#### Pericyte dysfunction and microvascular destabilization in the ApoE4 context

Pericytes, critical regulators of BBB function and microvascular tone, are increasingly recognized as central players in cerebrovascular pathophysiology [28, 94]. Evidence suggests that ApoE4 impairs pericyte function through both inflammatory and metabolic pathways, contributing to vascular fragility in the face of ischemic or hypoxic insult.

Halliday et al., [40] showed that pericyte degeneration is significantly more pronounced in APOE4 carriers with AD than in APOE3 carriers or non-AD controls. This loss was associated with increased fibrinogen and IgG leakage into brain parenchyma. Importantly, pericytes in these brains exhibited upregulated CypA and MMP-9 expression and reduced LRP1, indicating that the same CypA–NF-κB–MMP9 pathway implicated in endothelial dysfunction also impairs pericyte viability [40] (Fig. 4A-B). Pericyte dropout has been shown in stroke models to exacerbate BBB leakage and limit capillary reperfusion [30], suggesting that ApoE4 may worsen ischemic outcomes by promoting early microvascular collapse.

This was confirmed in vitro by Yamazaki et al., [93], who found that ApoE4-expressing pericytes impaired endothelial tube formation and failed to support barrier integrity. In co-culture assays, ApoE4 pericytes reduced TEER and decreased the expression of key extracellular matrix proteins, such as collagen IV, necessary for basement membrane stability. These deficits were intrinsic to ApoE4 pericytes, as endothelial ApoE genotype had no effect. In vivo, ApoE4 mice exhibited thinner basement membranes and fragmented capillaries, features commonly seen after cerebral ischemia [93].

Another important study by Blanchard et al., [14] used an iPSC-derived human BBB model to show that ApoE4-expressing pericytes promote vascular amyloid deposition via hyperactivation of the calcineurin (CaN)–NFAT pathway. Although the focus was on amyloid pathology, the upregulation of ApoE expression and pericyte-mediated barrier dysfunction observed here mirrors molecular patterns seen in stroke and chronic hypoperfusion models. Notably, CaN inhibition reduced both ApoE4-driven barrier impairment and vascular amyloid, indicating that modulating pericyte signaling pathways may improve vascular resilience [14].

#### Neuroinflammation and cytokine response

Among the three major human ApoE isoforms, the ε4 allele is consistently associated with heightened inflammatory responses in the CNS, which may exacerbate injury and impair recovery in the context of stroke, hypoxia-ischemia, or hemorrhage.

**Fig. 5:**
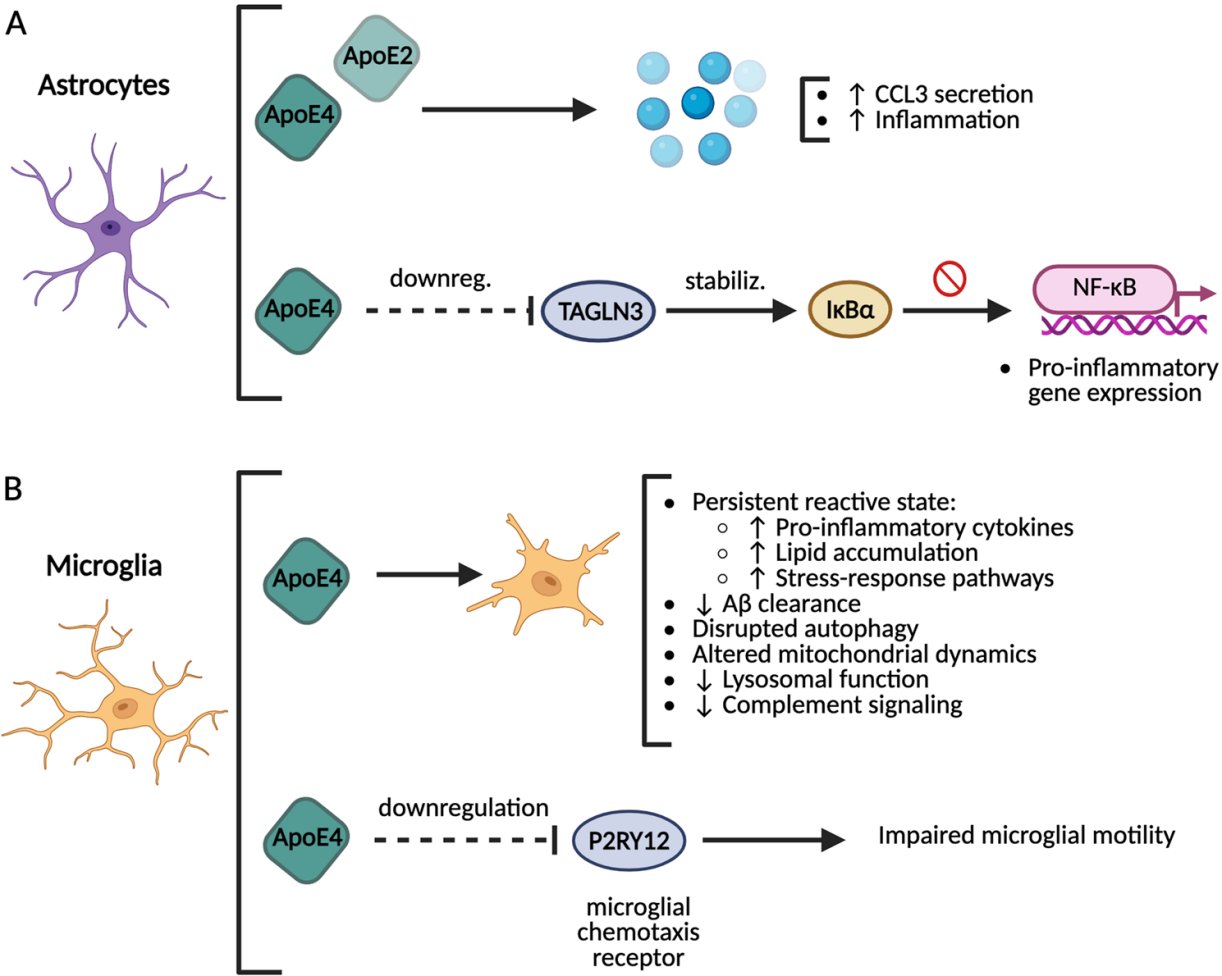
Molecular mechanisms linking ApoE ε4 and neuroinflammation. **A** In astrocytes, ApoE ε2 and ε4 show increased secretion of the microglial chemoattractant CCL3 upon TLR stimulation, which results in an increased inflammatory state. Furthermore, ApoE ε4 downregulates TAGLN3, protein that stabilizes the NF-kB inhibitor IkBα, resulting in an increased pro-inflammatory gene extression. **B** ApoE ε4 microglia are characterized for a persistent reactive state, reduced Aβ clearance, disrupted autophagy, altered mitochondrial dynamics, and reduced lysosomal function and complement signaling. In addition, ApoE ε4 downregulates the microglial chemotaxis receptor P2RY12, causing impaired microglial motility

One of the earliest indications of genotype-dependent inflammatory modulation came from the study by Cudaback et al., [23], who demonstrated that astrocytes derived from ApoE-targeted replacement (ApoE-TR) mice secreted significantly higher levels of the microglial chemoattractant CCL3 upon stimulation with Toll-like receptor (TLR) ligands in ApoE2 and ApoE4 cells compared to ApoE3 (Fig. 5A). These in vitro findings were corroborated in vivo, where ApoE4-TR mice exhibited elevated CCL3 levels following systemic lipopolysaccharide (LPS) exposure. Importantly, postmortem analysis of human parietal cortex tissue from AD patients revealed increased CCL3 expression in individuals homozygous for the APOE4 allele, highlighting the clinical relevance of these observations [23]. Given that CCL3 is a key chemokine involved in microglial recruitment and activation, its overexpression in ApoE4 carriers could facilitate the propagation of neuroinflammatory cascades during cerebrovascular injury.

Further mechanistic insight was provided by Arnaud et al., [4], who used isogenic human induced pluripotent stem cell (iPSC)-derived astrocytes differing only in APOE genotype to explore genotype-specific inflammatory signaling. Astrocytes homozygous for APOE4 exhibited elevated basal inflammation and exaggerated responses to pro-inflammatory stimuli such as TNF-α. This phenotype was mechanistically linked to the downregulation of transgelin 3 (TAGLN3), a protein that stabilizes IκBα, the cytoplasmic inhibitor of the NF-κB pathway. Loss of TAGLN3 in APOE4 astrocytes resulted in accelerated IκBα degradation and enhanced NF-κB nuclear translocation, thereby sustaining pro-inflammatory gene expression (Fig. 5A). Notably, decreased TAGLN3 expression was also observed in APOE4-positive postmortem AD’s brains, and restoration of TAGLN3 in vitro was sufficient to normalize inflammatory responses, positioning the ApoE4–TAGLN3–NF-κB axis as a central driver of astrocyte-mediated neuroinflammation [4]. In the context of cerebrovascular injury, especially during the perinatal period where glial plasticity is essential [27], such chronic astrocytic priming could worsen tissue damage and hinder repair.

Microglia, as the resident immune cells of the CNS, are also substantially affected by APOE genotype. Machlovi et al., [55] demonstrated that microglia from ApoE4-expressing humanized mice exhibit a chronically dysregulated phenotype, characterized by elevated levels of pro-inflammatory cytokines such as TNF-α and CXCL2, increased lipid accumulation, and markers of cellular stress (Fig. 5B). Although these microglia showed enhanced phagocytic uptake of particles including myelin and apoptotic cells, they displayed impaired degradation capacity, leading to sustained inflammation and lipid overload. Transcriptomic profiling revealed upregulated interferon signaling and chronic immune activation, suggesting that ApoE4 primes microglia toward a persistently reactive state [55]. Such a pro-inflammatory microglial phenotype could amplify secondary injury cascades following hypoxia-ischemia or hemorrhage, particularly in the immature brain.

Adding to this, Sepulveda et al., [80] showed that ApoE4 significantly impairs microglial motility, a critical feature for effective surveillance and response to injury. Using ex vivo brain slices from ApoE3 and ApoE4 knock-in mice, they found that ApoE4 microglia exhibited reduced chemotactic responses to injury (induced by the damage-associated signal ATP or injection of fibrillar Aβ). This was linked to translational downregulation of the P2RY12 receptor, essential for microglial chemotaxis, without corresponding decreases in mRNA levels (Fig. 5B) [80]. As microglial migration toward injury is a key step in limiting tissue damage and facilitating debris clearance [43], ApoE4-associated impairments in this function likely contribute to prolonged inflammation and poor outcomes in cerebrovascular contexts.

In addition to functional and chemotactic deficits, APOE4 microglia also suffer from impaired intracellular homeostasis. Bassal et al., [8] reported that ApoE4-expressing microglia, in addition to impaired clearance of Aβ, also display disrupted autophagic flux, and altered mitochondrial dynamics, including diminished membrane potential and defective mitophagy (Fig. 5B). These mitochondrial impairments were reversible with treatment with the autophagy inducer rapamycin, suggesting deficits in autophagy to be the cause of the observed mitochondrial dysfunction. The described dysfunctional organelle turnover and bioenergetic stress may likely be equally detrimental in perinatal cerebrovascular injury, where metabolic resilience is vital for cell survival and repair [8].

Extending these findings at the levels of brain function, Tao et al., [84] showed that higher cerebrospinal fluid (CSF) levels of the pro-inflammatory cytokine TNF-α were associated with reduced functional connectivity (FC) in cognitively critical brain regions. This relationship was even stronger in individuals carrying the APOE4 allele, suggesting that ApoE4 may sensitize the brain to inflammatory disruption of network-level function. While this study focused on older adults, similar mechanisms may impair circuit development and cognitive outcomes following early-life cerebrovascular events in ApoE4 carriers [84].

The functional implications of these inflammatory mechanisms were further illustrated by Liu et al., [54], who used an amyloid murine model in combination with transgenic mice expressing human ApoE isoforms specifically in microglia and CNS-associated macrophages to investigate how APOE genotype shapes microglial function. Mice expressing ApoE4 displayed impaired lipid metabolism, reduced phagocytic ability, and compromised immune activation, leading to increased amyloid deposition and exacerbated neuroinflammation (Fig. 5B). Single-cell transcriptomics confirmed that ApoE4-expressing microglia downregulated genes related to lysosomal function and complement signaling while upregulating stress-response pathways. These findings were reproduced in postmortem human AD brains, where APOE4-associated microglia accumulated lipid droplets and showed reduced capacity to engage with amyloid plaques. Although conducted in an AD’s model, these findings emphasize a generalized microglial dysfunction under ApoE4 that could impair resolution of inflammation following cerebrovascular injury [54].

Lastly, the interaction between neurons and glia under the influence of APOE genotype also plays a significant role in shaping the inflammatory landscape. Rao et al., [74] demonstrated that neuronal expression of ApoE4 drives a distinct neuroinflammatory response via microglial activation. After developing a chimeric AD’s model in which human iPSC-derived neurons homozygotic for APOE4, APOE3, or APOE knockout were transplanted into the hippocampus of ApoE-TR mice homozygotic for ApoE3 of ApoE4, it was observed that neuronal ApoE4 induced greater amyloid and tau pathology compared to APOE3 or APOE deficiency. Crucially, depletion of microglia significantly reduced these pathologies, confirming their central role in mediating ApoE4-driven neurotoxicity. Single-cell RNA sequencing revealed that microglia exposed to APOE4 neurons were shifted toward pro-inflammatory subtypes with increased expression of Major Histocompatibility Complex class II (MHC-II) genes. Although focused on neurodegeneration [74], this neuron-to-microglia inflammatory axis may also contribute to worsened outcomes in cerebrovascular injury, where neuronal stress can activate glial responses [91].

In summary, the ApoE4 genotype is associated with a complex array of molecular and cellular changes that collectively promote a pro-inflammatory environment in the brain. These include exaggerated cytokine production by astrocytes, chronic microglial activation and impaired function, disrupted mitochondrial and lysosomal pathways, and dysfunctional neuron-glia interactions. While most studies to date have been conducted in adult or aging models, the mechanistic pathways they reveal may be also relevant to perinatal cerebrovascular injury. The immature brain is particularly vulnerable to inflammation [39], and ApoE4-driven dysregulation of glial responses may significantly increase the severity of injury and impair long-term recovery.

#### ApoE4-induced impaired ß-amyloid clearance

It is important to acknowledge that β-amyloid (Aβ) accumulation is primarily studied in the context of aging and Alzheimer’s disease (AD), where it represents a central pathological hallmark [79]. In contrast, Aβ deposition in healthy individuals follows an age-dependent increase that is detectable in middle and older adulthood rather than during early development [92], and its relevance to the neonatal brain therefore remains uncertain. Accordingly, the extent to which Aβ clearance mechanisms contribute to PBI is currently unclear. Moreover, Aβ accumulation and clearance have not been systematically characterized in neonates or neonatal animal models, and whether Aβ plays a pathological role during this developmental window remains largely unexplored.

Aβ accumulation in the vasculature is strongly associated with vascular dysfunction and cognitive decline [2]. The distinct ApoE isoforms are known to affect Aβ clearance mechanisms [5], therefore the role of ApoE in the clearance of Aβ is another critical factor linking the ApoE genotype to the incidence and severity of cerebrovascular events like stroke, hypoxia-ischemia, and brain hemorrhages.

Several studies have demonstrated that the perivascular clearance of Aβ is impaired in models expressing the ApoE4 isoform. For instance, Hawkes et al., [41] observed that transgenic mice expressing human ApoE4 exhibited focal aggregates of Aβ along the brain vasculature, which were not observed in wild-type or ApoE3 mice. These aggregates were detected in both young and older ApoE4 mice but exacerbated by age. The impairment in Aβ clearance was linked to changes in the composition of the basement membrane in ApoE4 mice. Increased collagen IV and laminin levels at three months of age were followed by a decrease in these proteins at 16 months, which could hinder proper clearance and facilitate Aβ accumulation [41]. This alteration in the basement membrane structure, potentially driven by ApoE4-related processes, could promote vascular deposition of Aβ, contributing to neurovascular dysfunction.

The molecular mechanisms by which ApoE influences Aβ clearance are complex and involve interactions with various clearance receptors, including LRP1, which is essential for efficient Aβ efflux across the BBB [72]. Rolyan et al., [77] demonstrated that the source of ApoE (whether from astrocytes or neurons) affects its normal localization and its ability to interact with Aβ. While astrocyte-derived ApoE can autonomously drain into the perivascular space, neuronal ApoE only does that when forming a complex with Aβ. Those neuronal ApoE-Aβ complexes are then internalized via LRP1 receptor by astrocytes in order to access the perivascular space [77].The altered trafficking of neuronal ApoE and Aβ complexes could explain the observed deficits in Aβ clearance in ApoE4 carriers, as Aβ may be rerouted to less efficient clearance pathways, such as those involving the very low-density lipoprotein receptor (VLDLR), rather than the faster LRP1 route [25].

ApoE isoform-specific effects on Aβ clearance have been further elucidated by Castellano et al., [17], who demonstrated that while the synthesis of Aβ is unaffected by ApoE isoforms, the clearance rates of Aβ vary significantly. ApoE4 carriers displayed the slowest Aβ clearance rates, a phenomenon that worsens with age. This impaired clearance in ApoE4 mice is likely driven by inefficient receptor-mediated efflux, further compounding the risk of Aβ accumulation and vascular dysfunction over time [17]. Deane et al., [25] also confirmed that ApoE4 redirects Aβ to slower clearance pathways, reducing the overall efficacy of Aβ efflux and potentially promoting amyloid angiopathy, a condition characterized by the accumulation of Aβ in the brain vasculature that can lead to cerebral hemorrhage and other cerebrovascular events.

Interestingly, studies have also shown that ApoE may not only affect central Aβ clearance but also influence peripheral Aβ clearance. Hone et al., [45] demonstrated that ApoE is critical for the peripheral clearance of Aβ, as ApoE-knockout mice failed to clear intravenously injected Aβ42 through the liver and kidneys, leading to an accumulation of Aβ in the central nervous system. This impaired peripheral clearance could exacerbate central Aβ deposition, and may be particularly in ApoE4 carriers, where inefficient Aβ clearance pathways could increase the bioavailability of Aβ in the brain, potentially accelerating vascular deposition and impairing vascular integrity [45].

Recent studies also link lower levels of peripheral Aβ with accumulation of Aβ in the CNS. Blömeke et al., [15] developed a highly sensitive assay to quantify Aβ oligomers (an early toxic form of Aβ involved in AD) in plasma and observed that individuals with ApoE genotype, as well as mild cognitive impairment (MCI) or AD, exhibited reduced levels of Aβ oligomers in their plasma. The authors hypothesized that Aβ oligomers, once deposited into plaques, are cleared less efficiently from the peripheral circulation, which could contribute to the lower plasma levels observed in these individuals [15]. This reduction in peripheral Aβ clearance further reinforces the central role of ApoE in the overall clearance dynamics of Aβ, where inefficient clearance, particularly in ApoE4 carriers, may contribute to the pathogenesis of vascular and neurodegenerative diseases.

Although Aβ accumulation and clearance are not widely studied in the perinatal brain, this area could be important, as seen with tau phosphorylation, which is physiologically elevated in the developing brain of preterm and newborn infants [34]. However, a direct interaction between Aβ and tau has not been demonstrated in neonates. This suggests that studying Aβ clearance in the perinatal brain, particularly in relation to tau, could offer valuable insights into neurovascular integrity and developmental brain injury.

## Conclusion

PBI remains a leading cause of neonatal mortality and long-term neurodevelopmental impairment worldwide, yet its genetic determinants are still poorly understood. Among candidate genetic factors, the ApoE genotype has emerged as a potential modifier of susceptibility and outcome after perinatal cerebrovascular events. While most early studies yielded inconclusive or non-significant results, a recent large multicenter cohort study reported associations between ApoE ε2 and ε4 alleles and increased risk of severe IVH and adverse long-term outcomes such as cerebral palsy [26, 46]. These finding, however, require confirmation in independent cohorts. Experimental studies in animal and cell models further support a mechanistic role for ApoE in lipid metabolism, blood–brain barrier function, glial activation, and neuronal survival after hypoxic-ischemic injury, although most of these data derive from adult or non-perinatal contexts [19, 51, 60, 82].

Despite these advances, critical knowledge gaps remain. The precise isoform-specific mechanisms by which ApoE influences cerebrovascular vulnerability in the developing brain are still unclear, and most mechanistic insights come from adult or neurodegenerative contexts. Importantly, it remains unknown to what extent these pathways operate in the immature brain. Given the unique vulnerabilities of the developing brain, it is essential to determine whether ApoE-driven mechanisms identified in adult injury or neurodegeneration are conserved in neonates or whether distinct, developmentally specific processes are involved.

Future studies should focus on integrating genetic, molecular, and clinical data to clarify the contribution of ApoE genotype to perinatal outcomes. In particular, prospective multi-center cohorts with genomic stratification, combined with translational research in neonatal models, are needed to rigorously evaluate ApoE as a predictive biomarker. If future independent studies confirm these associations, ApoE genotyping may contribute to risk stratification approaches to identify infants at increased vulnerability to severe PBI. Although still speculative, ApoE-targeted strategies; such as modulation of lipid transport, inflammatory signaling, or vascular integrity; may eventually contribute to the development of neuroprotective interventions tailored to the perinatal period.

## Supplementary Information

Supplementary Material 1.

## Data Availability

No datasets were generated or analysed during the current study.

## Abbreviations

ABCA1: ATP-binding cassette transporter A1
Aβ: Amyloid-beta
AD: Alzheimer’s Disease
ApoE: Apolipoprotein E
ApoE-TR: Apolipoprotein E Targeted Replacement
BBB: Blood-brain barrier
CaN: Calcineurin
CGNs: Cerebellar Granule Neurons
CNS: Central Nervous System
CP: Cerebral Palsy
CSF: Cerebrospinal Fluid
CypA: Cyclophilin A
FA: Fatty Acids
FC: Functional Connectivity
GM-IVH: Germinal Matrix-Intraventricular Hemorrhage
HDL: High Density Lipoprotein
hFNSC: human Fetal Neural Stem Cells
HI: Hypoxia-ischemia
HIE: Hypoxic-ischemic Encephalopathy
HII: Hypoxic-ischemic Injury
HSPG: Heparan Sulphate Proteoglycans
ICV: Intracerebroventricular
iPSC: induced Pluripotent Stem Cells
KO: Knockout
LD: Lipid Droplets
LDL: Low Density Liporprotein
LDLR: Low Density Lipoprotein Receptor
LPS: Lipopolysaccharide
LRP1: Low Density Lipoprotein Receptor-Related Protein 1
MCI: Mild Cognitive Impairment
MHC-II: Major Histocompatibility Complex Class II
NMDA: N-methyl-D-aspartic-acid
OGDR: Oxygen Glucose Deprivation Reperfusion
PBI: Perinatal Brain Injury
PKCη: Protein Kinase C eta
RNAi: RNA interference
TAGLN3: Transgelin-3
TEER: Transendothelial Electrical Resistance
TLR: Toll-like Receptor
VLDL: Very-Low-Density Lipoprotein
VLDLR: Very-Low-Density Lipoprotein Receptor
WMI: White Matter Injury
