# Supplementary material for: Is there a role for apolipoprotein E in perinatal brain injury?
Source: Mol Cell Pediatr. 2026 Mar 3;13:9. doi: 10.1186/s40348-026-00223-6 (PMC12953799; doi:10.1186/s40348-026-00223-6)
Supplement: Supplementary file 1 — Supplementary Material 1. [file 40348_2026_223_MOESM1_ESM.docx]

**Annex 1.** **Studies screened for inclusion in the systematic review**. Overview of all records identified and screened in the systematic literature search conducted for Section 4 of this review. The table lists the first author and year, full citation, inclusion or exclusion status, and the primary reason for exclusion where applicable. Studies were excluded if they were not related to perinatal brain injury, did not assess ApoE genotype, were experimental or review articles, or represented commentaries on previously published studies. The final set of included studies corresponds to those summarized in the qualitative synthesis and reported in the PRISMA flow diagram.

| **First author and year** | **Citation** | **Included/**  **Excluded** | **Exclusion reason** |
| --- | --- | --- | --- |
| Artieda et al. (2008) | Artieda M, Gañán A, Cenarro A, García-Otín AL, Jericó I, Civeira F, Pocoví M. Association and linkage disequilibrium analyses of APOE polymorphisms in atherosclerosis. Dis Markers. 2008;24(2):65-72. doi: 10.1155/2008/650410. PMID: 18219091; PMCID: PMC3850622. | Excluded | Not related to PBI |
| Balcerzyk et al. (2010) | Balcerzyk A, Zak I, Niemiec P, Kopyta I, Emich-Widera E, Iwanicki T, Pilarska E, Pienczk-Recławowicz K, Kacinski M, Wendorff J, Jachowicz-Jeszka J. APOE gene epsilon polymorphism does not determine predisposition to ischemic stroke in children. Pediatr Neurol. 2010 Jul;43(1):25-8. doi: 10.1016/j.pediatrneurol.2010.02.016. Erratum in: Pediatr Neurol. 2010 Nov;43(5):374. Pilarska, Ewa [added]; Pienczk-Recławowicz, Karolina [added]; Kacinski, Marek [added]; Wendorff, Jerzy [added]; Jachowicz-Jeszka, Joanna [added]. PMID: 20682199. | Included |  |
| Becher et al. (2006) | Becher JC, Keeling JW, McIntosh N, Wyatt B, Bell J. The distribution of apolipoprotein E alleles in Scottish perinatal deaths. J Med Genet. 2006 May;43(5):414-8. doi: 10.1136/jmg.2005.033936. Epub 2005 Sep 23. PMID: 16183800; PMCID: PMC2564516. | Included |  |
| Bell et al. (2015) | Bell JE, Becher JC, Keeling JW, McIntosh N. The neuropathology of stillbirth - correlation with apolipoprotein genotype in a Scottish population based study. Early Hum Dev. 2015 Feb;91(2):139-48. doi: 10.1016/j.earlhumdev.2014.12.008. Epub 2015 Jan 22. PMID: 25617864. | Excluded | Not related to PBI |
| Blackman et al. (2005) | Blackman JA, Worley G, Strittmatter WJ. Apolipoprotein E and brain injury: implications for children. Dev Med Child Neurol. 2005 Jan;47(1):64-70. doi: 10.1017/s0012162205000113. PMID: 15686292. | Excluded | Not related to PBI (traumatic brain injury) |
| Blackman et al. (2009) | Blackman JA, Gurka MJ, Bao Y, Dragulev BP, Chen WM, Romness MJ. Apolipoprotein E and functional motor severity in cerebral palsy. J Pediatr Rehabil Med. 2009;2(1):67-74. doi: 10.3233/PRM-2009-0063. PMID: 21791796. | Included |  |
| Blackman et al. (2014) | Blackman JA, Gordish-Dressman H, Bao Y, Matsumoto JA, Sinkin RA. The apolipoprotein gene and recovery from brain injury among extremely preterm infants. Neonatology. 2014;105(3):227-9. doi: 10.1159/000357700. Epub 2014 Feb 6. PMID: 24503939; PMCID: PMC4030425. | Included |  |
| Chen et al. (2024) | Chen H, Zhao S, Jian Q, Yan Y, Wang S, Zhang X, Ji Y. The role of ApoE in fatty acid transport from neurons to astrocytes under ischemia/hypoxia conditions. Mol Biol Rep. 2024 Feb 23;51(1):320. doi: 10.1007/s11033-023-08921-4. PMID: 38393618. | Excluded | Experimental study |
| Coen Herak et al. (2017) | Coen Herak D, Lenicek Krleza J, Radic Antolic M, Horvat I, Djuranovic V, Zrinski Topic R, Zadro R. Association of Polymorphisms in Coagulation Factor Genes and Enzymes of Homocysteine Metabolism With Arterial Ischemic Stroke in Children. Clin Appl Thromb Hemost. 2017 Nov;23(8):1042-1051. doi: 10.1177/1076029616672584. Epub 2016 Oct 18. PMID: 28301901. | Included |  |
| Cotten et al. (2014) | Cotten CM, Goldstein RF, McDonald SA, Goldberg RN, Salhab WA, Carlo WA, Tyson JE, Finer NN, Walsh MC, Ehrenkranz RA, Laptook AR, Guillet R, Schibler K, Van Meurs KP, Poindexter BB, Stoll BJ, O'Shea TM, Duara S, Das A, Higgins RD, Shankaran S; Eunice Kennedy Shriver National Institute of Child Health and Human Development Neonatal Research Network. Apolipoprotein E genotype and outcome in infants with hypoxic-ischemic encephalopathy. Pediatr Res. 2014 Mar;75(3):424-30. doi: 10.1038/pr.2013.235. Epub 2013 Dec 9. PMID: 24322171; PMCID: PMC4095992. | Included |  |
| Dzietko et al. (2019) | Dzietko M, Schulz S, Preuss M, Haertel C, Stein A, Felderhoff-Mueser U, Goepel W. Apolipoprotein E gene polymorphisms and intraventricular haemorrhage in infants born preterm: a large prospective multicentre cohort study. Dev Med Child Neurol. 2019 Mar;61(3):337-342. doi: 10.1111/dmcn.13987. Epub 2018 Aug 7. PMID: 30084487. | Included |  |
| Gelfand et al. (2013) | Gelfand AA, Croen LA, Torres AR, Wu YW. Genetic risk factors for perinatal arterial ischemic stroke. Pediatr Neurol. 2013 Jan;48(1):36-41. doi: 10.1016/j.pediatrneurol.2012.09.016. PMID: 23290018; PMCID: PMC3539155. | Included |  |
| Humberg et al. (2022) | Humberg A, Dzietko M, Schulz S, Felderhoff-Müser U, Hüning B, Stein A, Fortmann MI, Marissen J, Rausch TK, Herting E, Härtel C, Göpel W; German Neonatal Network (GNN). Association of ApoE Genotypes and Recovery From Intracerebral Hemorrhage in Very Low Birth Weight Infants. Stroke. 2022 Feb;53(2):514-522. doi: 10.1161/STROKEAHA.120.033432. Epub 2021 Oct 8. PMID: 34619984. | Included |  |
| Kasapkara et al. (2013) | Kasapkara ÇS, Tümer L, Biberoglu G, Kasapkara A, Hasanoğlu A. Asymmetric dimethylarginine (ADMA) and L-arginine levels in children with glycogen storage disease type I. J Pediatr Endocrinol Metab. 2013;26(5-6):427-31. doi: 10.1515/jpem-2012-0306. PMID: 23412857. | Excluded | Not related to ApoE or PBI |
| Korja et al. (2013) | Korja M, Ylijoki M, Lapinleimu H, Pohjola P, Matomäki J, Kuśmierek H, Mahlman M, Rikalainen H, Parkkola R, Kaukola T, Lehtonen L, Hallman M, Haataja L. Apolipoprotein E, brain injury and neurodevelopmental outcome of children. Genes Brain Behav. 2013 Apr;12(3):348-52. doi: 10.1111/gbb.12024. Epub 2013 Mar 7. PMID: 23387365. | Included |  |
| Krleza et al. (2023) | Krleza JL, Coen Herak D, Đakovic I, Vulin K, Roic G, Tripalo Batoš A, Čeri A, Zadro R, Đuranovic V. Inherited Thrombophilia Associated With Ischemic Pediatric Stroke in Parent-Child Pairs. Pediatr Neurol. 2023 Sep;146:119-128. doi: 10.1016/j.pediatrneurol.2023.06.017. Epub 2023 Jun 28. PMID: 37480820. | Excluded | Not related to ApoE genotype |
| Kuroda et al. (2007) | Kuroda MM, Weck ME, Sarwark JF, Hamidullah A, Wainwright MS. Association of apolipoprotein E genotype and cerebral palsy in children. Pediatrics. 2007 Feb;119(2):306-13. doi: 10.1542/peds.2006-1083. PMID: 17272620. | Excluded | Not related to PBI |
| Lal et al. (2012) | Lal C, Strange C, Bachman D. Neurocognitive impairment in obstructive sleep apnea. Chest. 2012 Jun;141(6):1601-1610. doi: 10.1378/chest.11-2214. PMID: 22670023. | Excluded | Review study |
| Lendon et al. (2000) | Lendon CL, Han BH, Salimi K, Fagan AM, Behrens MI, Muller MC, Holtzman DM. No effect of apolipoprotein E on neuronal cell death due to excitotoxic and apoptotic agents in vitro and neonatal hypoxic ischaemia in vivo. Eur J Neurosci. 2000 Jul;12(7):2235-42. doi: 10.1046/j.1460-9568.2000.00113.x. PMID: 10947802. | Excluded | Experimental study |
| Lombardi et al. (1998) | Lombardi VR, García M, Cacabelos R. Microglial activation induced by factor(s) contained in sera from Alzheimer-related ApoE genotypes. J Neurosci Res. 1998 Nov 15;54(4):539-53. doi: 10.1002/(SICI)1097-4547(19981115)54:4<539::AID-JNR11>3.0.CO;2-Q. PMID: 9822164. | Excluded | Not related to PBI |
| McNeil (2005) | McNeill A. Expression of apolipoprotein-E in human perinatal brain after hypoxic-ischaemic injury. Pathology. 2005 Jun;37(3):256-8. doi: 10.1080/00313020500099007. PMID: 16175903. | Included |  |
| Moceri et al. (2000) | Moceri VM, Kukull WA, Emanuel I, van Belle G, Larson EB. Early-life risk factors and the development of Alzheimer's disease. Neurology. 2000 Jan 25;54(2):415-20. doi: 10.1212/wnl.54.2.415. PMID: 10668705. | Excluded | Not related to PBI |
| Oriá et al. (2005) | Oriá RB, Patrick PD, Zhang H, Lorntz B, de Castro Costa CM, Brito GA, Barrett LJ, Lima AA, Guerrant RL. APOE4 protects the cognitive development in children with heavy diarrhea burdens in Northeast Brazil. Pediatr Res. 2005 Feb;57(2):310-6. doi: 10.1203/01.PDR.0000148719.82468.CA. Epub 2004 Dec 20. PMID: 15611352. | Excluded | Not related to PBI |
| Oriá et al. (2023) | Oriá RB, Freitas RS, Roque CR, Nascimento JCR, Silva AP, Malva JO, Guerrant RL, Vitek MP. ApoE Mimetic Peptides to Improve the Vicious Cycle of Malnutrition and Enteric Infections by Targeting the Intestinal and Blood-Brain Barriers. Pharmaceutics. 2023 Mar 28;15(4):1086. doi: 10.3390/pharmaceutics15041086. PMID: 37111572; PMCID: PMC10141726. | Excluded | Not related to PBI |
| Rotstein et al. (2006) | Rotstein M, Bassan H, Kariv N, Speiser Z, Harel S, Gozes I. NAP enhances neurodevelopment of newborn apolipoprotein E-deficient mice subjected to hypoxia. J Pharmacol Exp Ther. 2006 Oct;319(1):332-9. doi: 10.1124/jpet.106.106898. Epub 2006 Jul 5. Erratum in: J Pharmacol Exp Ther. 2007 Jan;320(1):498. PMID: 16822898. | Excluded | Experimental study |
| Sahu (2011) | Sahu JK. Association between APOE gene ɛ polymorphism and ischemic stroke in children. Pediatr Neurol. 2011 Jan;44(1):80; author reply 80-1. doi: 10.1016/j.pediatrneurol.2010.09.004. PMID: 21147399. | Excluded | Commentary on another study (Belcerzyk et al. 2010) |
| Seymour et al. (2007) | Seymour GJ, Ford PJ, Cullinan MP, Leishman S, Yamazaki K. Relationship between periodontal infections and systemic disease. Clin Microbiol Infect. 2007 Oct;13 Suppl 4:3-10. doi: 10.1111/j.1469-0691.2007.01798.x. PMID: 17716290. | Excluded | Not related to ApoE or PBI |
| Shrivastava et al. (2024) | Shrivastava V, Tyagi S, Dey D, Singh A, Palanichamy JK, Sinha S, Sharma JB, Seth P, Sen S. Glial cholesterol redistribution in hypoxic injury in vitro influences oligodendrocyte maturation and myelination. Biochim Biophys Acta Mol Basis Dis. 2024 Dec;1870(8):167476. doi: 10.1016/j.bbadis.2024.167476. Epub 2024 Aug 22. PMID: 39181517. | Excluded | Not related to ApoE genotype |
| Siegel et al. (2016) | Siegel G, Mockenhaupt FHME, Behnke AL, Ermilov E, Winkler K, Pries AR, Malmsten M, Hetzer R, Saunders R, Lindman B. Lipoprotein binding to anionic biopolyelectrolytes and the effect of glucose on nanoplaque formation in arteriosclerosis and Alzheimer's disease. Adv Colloid Interface Sci. 2016 Jun;232:25-35. doi: 10.1016/j.cis.2016.02.001. Epub 2016 Feb 19. PMID: 26969281. | Excluded | Not related to PBI |
| Topriceanu et al. (2024) | Topriceanu CC, Shah M, Webber M, Chan F, Shiwani H, Richards M, Schott J, Chaturvedi N, Moon JC, Hughes AD, Hingorani AD, O'Regan DP, Captur G. APOE ε4 carriage associates with improved myocardial performance from adolescence to older age. BMC Cardiovasc Disord. 2024 Mar 21;24(1):172. doi: 10.1186/s12872-024-03808-z. PMID: 38509472; PMCID: PMC10956279. | Excluded | Not related to PBI |
| Vollmer (2019) | Vollmer B. Apolipoprotein E gene polymorphisms: a risk factor for preterm brain injury? Dev Med Child Neurol. 2019 Mar;61(3):287. doi: 10.1111/dmcn.14061. Epub 2018 Oct 3. PMID: 30284238. | Excluded | Commentary on another study (Dzietko et al. 2019) |
| Zhang et al. (2011) | Zhang HL, Lei J, Gao SJ, Yang Y, Wu J. APOE gene ɛ polymorphism and ischemic stroke in children. Pediatr Neurol. 2011 Jan;44(1):79; author reply 79-80. doi: 10.1016/j.pediatrneurol.2010.09.006. PMID: 21147398. | Excluded | Commentary on another study (Belcerzyk et al. 2010) |
